# Pagetoid spread of urothelial carcinoma controlled without resection

**DOI:** 10.1002/iju5.12555

**Published:** 2022-11-06

**Authors:** Shutaro Yamamoto, Tatsuya Shimomura, Kanako Kasai, Taisuke Yamazaki, Yuki Enei, Yusuke Koike, Toshihiro Ito, Tohru Harada, Nozomu Furuta, Takahiro Kimura

**Affiliations:** ^1^ Department of Urology The Jikei University Daisan Hospital Komae Tokyo Japan; ^2^ Department of Urology JR Tokyo General Hospital Shibuya Tokyo Japan; ^3^ Department of Urology The Jikei University School of Medicine Minato Tokyo Japan; ^4^ Department of Dermatology The Jikei University Daisan Hospital Komae Tokyo Japan; ^5^ Department of Pathology The Jikei University Daisan Hospital Komae Tokyo Japan

**Keywords:** chemotherapy, extramammary Paget's disease, urinary bladder neoplasms, vulva, vulvectomy

## Abstract

**Introduction:**

Extramammary Paget's disease is an eczematous skin condition that affects the vulva and perineum. Extramammary Paget's disease secondary to urothelial carcinoma is a rare condition that is typically treated with invasive surgical resection of the lesion.

**Case presentation:**

An 80‐year‐old woman with a 7‐year history of urothelial carcinoma presented with erythema of the labia majora. Immunostaining of skin biopsy specimens suggested extramammary Paget's disease secondary to urothelial carcinoma. The patient did not consent to resection of the lesion. Nine cycles of first‐line platinum‐based chemotherapy for metastatic urothelial carcinoma were administered. As tumor cells remained after systemic chemotherapy, pembrolizumab will be administered to the patient for treating residual extramammary Paget's disease.

**Conclusion:**

Platinum‐based chemotherapy can control extramammary Paget's disease secondary to urothelial carcinoma.

**Abbreviations & Acronyms**
BCG: Bacillus Calmette‐Guerin
CIS: carcinoma *in situ*
CK20: cytokeratin 20
CK7: cytokeratin 7
EMPD: extramammary Paget's disease
GCDFP‐15: gross cystic disease fluid protein‐15‐negative
H&E: hematoxylin and eosin
HG: high‐grade
LG: low‐grade
ND: no data
NMIBC: non‐muscle invasive bladder cancer
PR: partial response
pTa: cancer grade pTa
pTis: cancer grade pTis
TCC: transitional cell carcinoma
TURBT: transurethral resection of bladder tumor
UC: urothelial carcinoma

**Keynote message**

Extramammary Paget's disease (EMPD) of the vulva secondary to urothelial carcinoma is rare. The standard of care for EMPD is vulvectomy. However, this procedure is highly invasive. Our findings suggest that chemotherapy might be an alternative to invasive surgery in secondary EMPD, resulting in higher patient satisfaction.

## Introduction

Paget's disease is a tumor of the sweat gland system that typically does not form a mass and is histopathologically characterized by the presence of large, faint Paget's cells. EMPD occurs outside the breast and was first reported by Crocker in 1889.^1^ EMPD is classified as a primary disease or as secondary to other carcinomas. The incidence of EMPD has been reported to be 0.12 per 100 000 individuals, and EMPD of the vulva accounts for 2% of all vulvar malignancies.^2^ Pagetoid spread is a rare phenomenon, in which UC adjacent to the skin develops in the epithelium and reaches the epidermis, resembling intraepidermal cancer. Surgical excision is the standard treatment for EMPD.

Few works have reported pagetoid spread of UC (Table 1). This case report describes a patient with EMPD secondary to UC who declined surgical resection and was administered systemic chemotherapy.

**Table 1:** Published case reports on EMPD secondary to UC and their details

Study	Number of cases	Age/sex	Primary	Pathology	Treatment for primary site	Time from initial treatment to EMPD diagnosis (year)	Other visceral metastasis/recurrence	Lymph node metastasis	Treatment for EMPD	Chemotherapy	Follow‐up	Outcome
Wilkinson *et al*.^3^ 2002	3	76/F	Bladder	CIS	BCG	18	Bladder carcinoma recurrence	ND	Vulvectomy	None	ND	ND
		81/F	Bladder	CIS	BCG	6	Vagina HG neoplasm, bladder CIS recurrence	None	Vulvectomy	ND	9 months	No recurrence
		76/F	ND	ND	ND	ND	Bladder carcinoma	ND	ND	ND	ND	ND
Salamanca *et al*.^4^ 2004	2	68/M	Bladder	CIS	BCG	Same time	None	None	ND	ND	14 months	Bladder TCC recurrence
		68/M	Bladder	CIS HG UC	Cystourethrectomy with lymphadenectomy	4	Liver metastasis	ND	None	None	3 months	Deceased
Brown *et al*.^5^ 2005	1	81/F	Bladder	CIS	ND	ND	Vagina HG	ND	Vulvectomy	ND	ND	ND
Kurashige *et al*.^6^ 2013	1	64/F	Bladder	HG UC	Cystectomy	3	Vagina, bone	Distal lymph nodes	Surgical treatment	ND	ND	ND
Kiyohara *et al*.^7^ 2013	1	72/F	Bladder	ND	Cystectomy	9	ND	ND	Urethrectomy	ND	ND	ND
Gulavita *et al*.^8^ 2014	1	71/M	Bladder	HG UC	TURBT	4	None	Inguinal lymph node	Hemiscrotectomy	Administered	6 months	Deceased
Fuentes *et al*.^9^ 2015	1	47/M	Bladder	CIS	Cystectomy	9	Urethral recurrence	ND	Urethrectomy	None	ND	ND
Qian *et al*.^10^ 2018	1	63/M	Right ureter	LG UC	Nephroureterectomy with partial cystectomy	5	None	None	ND	ND	2 years	Deceased for advanced bladder carcinoma
Chen *et al*.^11^ 2018	1	75/F	Bladder	infiltrative UC	ND	Same time	Lymph node metastasis and right obturator muscle	Details unknown	ND	ND	ND	ND
Nishikawa *et al*.^12^ 2019	1	75/M	Bladder, left ureter	pTis HG	Cystectomy, BCG for the left ureter	5	Urethral recurrence	None	Total penectomy	None	ND	ND
Primo *et al*.^13^ 2019	1	65/F	Bladder	NMIBC LG	TURBT, mitomycin, BCG	2	Cervical wall, uterine wall, vagina	Inguinal, pelvic, para‐aortic lymph node	Chemotherapy	Gemcitabine and cisplatin	6 cycles of chemotherapy	PR
Our case 2022	1	80/F	Bladder	UC pTa HG	TURBT, BCG	7	None	None	Chemotherapy	Gemcitabine and cisplatin	2 years	ND

## Case presentation

**Fig. 1:**
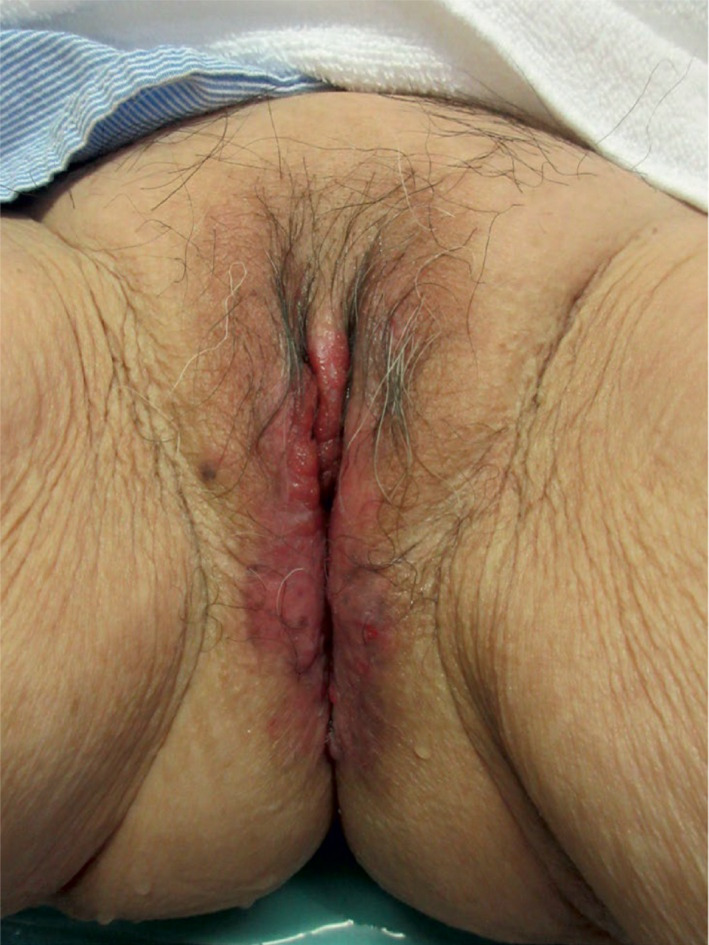
Erythema of labia majora.

An 80‐year‐old woman with a history of UC presented with painful erythema of the labia majora that began approximately 3 months earlier (Fig. 1). The lesion did not improve after using topical steroids and dimethyl isopropylazulene.

The patient had undergone transurethral resection of a HG (G2 > G3) UC (pTa) 7 years prior to her current presentation. Postoperatively, intravesical BCG was administered resulting in Reiter's syndrome symptoms. Then, urine cytology remained positive; however, no recurrence of UC was identified on cystoscopy, computed tomography, or magnetic resonance imaging. Transurethral biopsy and bilateral retrograde pyelogram were repeated three times over 2 years postoperatively, with no malignant tumors detected. The patient refused additional examinations thereafter.

**Fig. 2:**
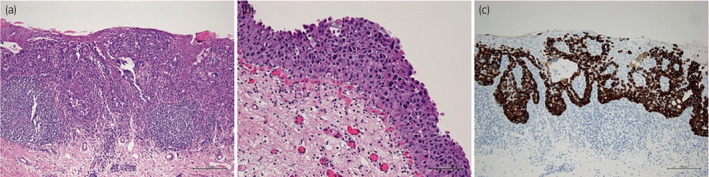
Biopsy specimen of erythematous skin. (a) The epidermis shows intense thickening owing to the proliferation of atypical cells in Paget's disease (H&E staining, magnification: 40×). (b) Pagetoid migration of atypical epithelial cells is observed near the granular layer. Some cells have clear cytoplasm (H&E staining, magnification: 400×). (c) Immunohistochemistry using anti‐CK20 antibodies reveals CK20‐positive cells, indicating EMPD.

The patient complained of painful erythema of the labia majora 7 years later. A skin biopsy performed by a dermatologist revealed non‐invasive secondary EMPD. The diagnosis was confirmed via immunostaining (CK20‐positive and GCDFP‐15) (Fig. 2).

Transurethral bladder biopsy and retrograde pyelography were conducted to determine the primary carcinoma. Bladder CIS was identified, and the cytologic sample from the left ureter was positive. No distant or lymph node metastasis was detected by precision imaging. No other suggestive lesions were observed on gynecological or gastroenterological examinations. The lesion was diagnosed as EMPD secondary to UC. Although the standard treatment for vulvar EMPD is vulvectomy, the patient did not consent to the operation. Intravesical BCG was contraindicated as she had previously developed Reiter's syndrome following BCG administration. Therefore, systemic chemotherapy was chosen, considering the UC (bladder and left upper urinary tract).

The patient was administered nine courses of platinum‐based chemotherapy (two courses of gemcitabine and cisplatin and seven courses of gemcitabine and carboplatin) for 18 months. The cisplatin combination regimen was changed to a carboplatin‐based regimen owing to declining renal function. During chemotherapy, the vulvar pain and erythema remained stable.

**Fig. 3:**
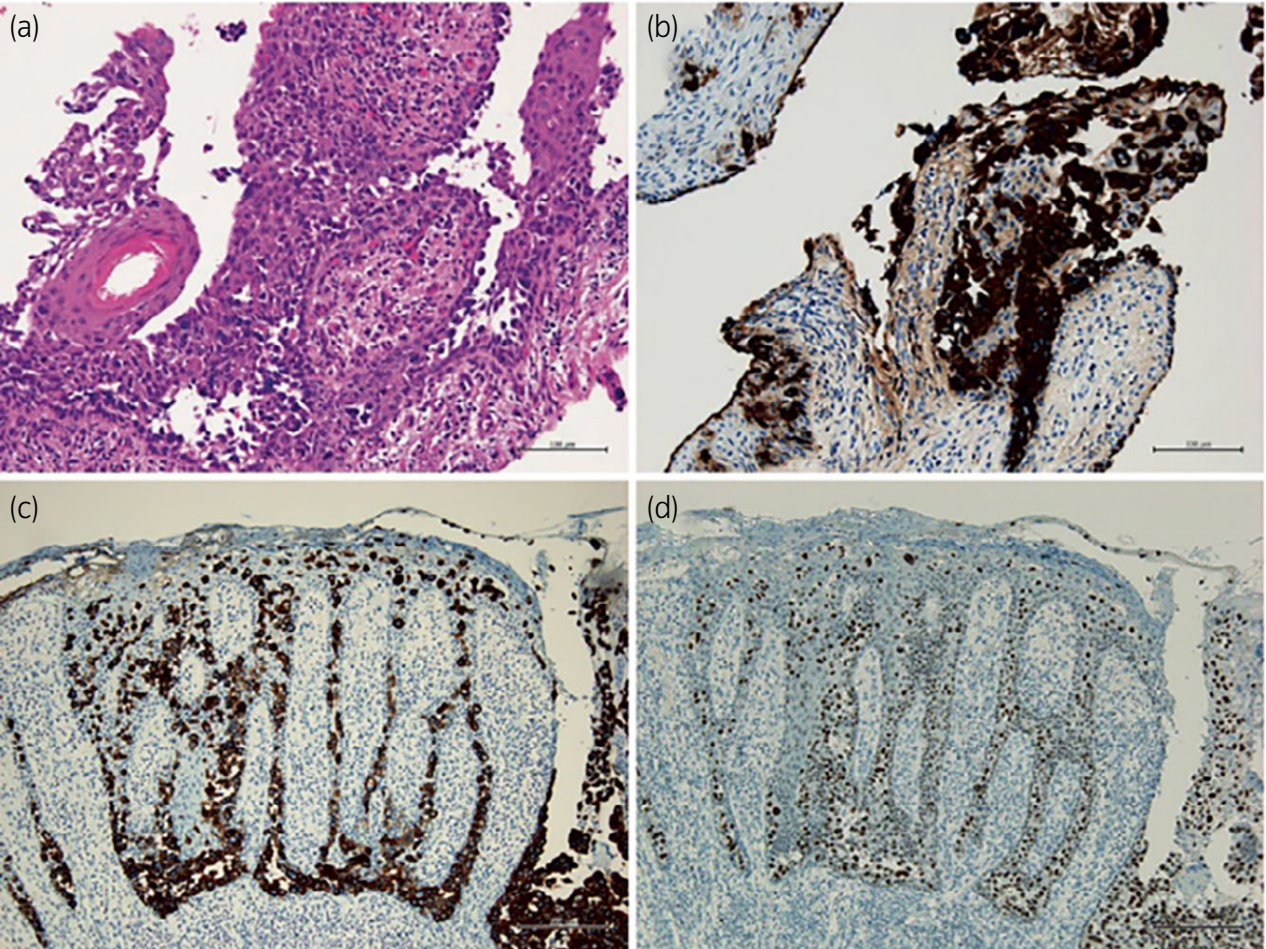
Biopsy specimen of erythematous skin. (a) H&E staining reveals Paget's cells. (b) Cells are positive for CK7, indicating secondary EMPD. (c) Cells are also positive for CK20, confirming secondary EMPD. (d) Cells are positive for GATA‐binding protein‐3, indicating urothelial cellular features.

Wedge resection of the erythema and a transurethral bladder biopsy, including retrograde pyelography, were conducted after chemotherapy to determine the patient's pathological outcome. No remarkable tumor or suggestive lesions were identified throughout the urinary tract. Immunostaining of the vulvar specimen was positive for CK20, CK‐7, and GATA‐binding protein 3, suggesting EMPD secondary to UC (Fig. 3). The specimen was unremarkable concerning possible histopathological effects of platinum‐based chemotherapy, such as protein denaturation or necrosis. As the lesion was not fully responsive to cisplatin‐based systemic chemotherapy, pembrolizumab was planned for the treatment of residual EMPD. On the last follow‐up examination 2 years after diagnosis, the patient showed no evidence of disease.

## Discussion

Initially, EMPD was diagnosed based on the presence of Paget's cells on the histopathological examination with H&E staining of lesion biopsy. For differential diagnosis, immunohistological analysis was useful. The usefulness of CK‐7, CK‐20, and GCDFP‐15 in EMPD diagnosis has been reported.^2^, ^3^, ^6^, ^7^, ^8^, ^9^, ^10^, ^11^, ^12^, ^13^, ^14^, ^15^ As CK‐7 is positive in primary and secondary EMPD, it could not be conclusive. Conversely, CK‐20 and GCDFP‐15 were useful in diagnosing secondary EMPD. Additional minute examination of the patient revealed no other malignant tumors except UC. Therefore, we diagnosed the lesion as EMPD secondary to UC.

Vulvar erythema was controlled by systemic chemotherapy without the need for surgical resection. Few studies on secondary EMPD are available, as most lesions are surgically resected.^14^ One reported case of EMPD secondary to UC with lymph node metastasis was treated without resection^13^; the patient underwent platinum‐based chemotherapy, with PR. Another report described the success of systemic chemotherapy in a patient with perianal EMPD secondary to anal canal carcinoma with liver and lymph node metastases.^15^ Although our patient had no metastasis other than vulvar Paget's disease, platinum‐based chemotherapy was administered addressing the primary cancer, and it appeared to have stabilized the EMPD erythema.

Few studies have been reported addressing the effectiveness of systemic chemotherapy targeting the primary cancer on a secondary EMPD.^8^, ^13^, ^15^ Systemic chemotherapy contributed to the treatment of EMPD in two previous reports,^13^, ^15^ possibly owing to the primary site and secondary EMPD sharing biological features. This was the rationale at the basis of our choice of first‐line treatment with a platinum‐based systemic chemotherapy regimen. It also motivated our choice for administrating pembrolizumab as the second‐line treatment. Additional research evidence is needed to confirm the effectiveness of systemic chemotherapy addressing the primary tumor in concomitant secondary EMPD.

Platinum‐based chemotherapy is the standard of care for advanced UC. Although chemotherapy is effective, most patients experience disease progression within 9 months: the median overall survival is 14–15 and 9–10 months after treatment with cisplatin‐based and carboplatin‐based regimens, respectively.^16^, ^17^, ^18^, ^19^, ^20^ Here, erythema did not exacerbate or progress, and the pain did not recur; thus, we concluded that it may effectively prevent the progression of secondary EMPD.

Previous case reports on EMPD secondary to UC published were reviewed (Table 1). The histological features of the primary UC may have contributed to patients' survival. CIS, a predictor of poor prognosis, is the most common primary pathological diagnosis. Here, the initial primary cancer diagnosis was HG (pTa) non‐invasive UC (no invasion of the muscle layer, which may have contributed to the favorable outcome). Few studies have described the long‐term outcomes of EMPD secondary to bladder cancer.

## Conclusion

In this patient, EMPD secondary to UC was controlled via nine cycles of platinum‐based chemotherapy. Thus, systemic chemotherapy might be an effective treatment for secondary EMPD and should be considered in lieu of invasive surgical resection. However, larger studies should verify this conclusion.

## Author contributions

Shutaro Yamamoto: Resources; writing – original draft. Tatsuya Shimomura: Supervision; writing – review and editing. Yuki Enei: Resources. Taisuke Yamazaki: Resources. Kanako Kasai: Resources. Yusuke Koike: Supervision; writing – review and editing. Toshihiro Ito: Resources. Tohru Harada: Resources. Nozomu Furuta: Supervision; writing – review and editing. Takahiro Kimura: Supervision; writing – original draft.

## Conflict of interest

The authors declare no conflict of interest.

## Approval of the research protocol by an Institutional Reviewer Board

Not applicable.

## Informed consent

Informed consent was obtained from the patient for the publication of this case report and the accompanying images.

## Registry and the Registration No. of the study/trial

Not applicable.
